# Fear of Negative Evaluation among College Students during the COVID-19 Pandemic: The Role of Adverse Childhood Experiences and Anxiety Sensitivity

**DOI:** 10.1007/s40653-025-00729-7

**Published:** 2025-08-18

**Authors:** Francesca A. St. Pe, Isabella K. Pallotto, Jacquana L. Smith, Angela Combs, Abigail McCarthy, Zoey Bass, Cathleen C. Odar Stough

**Affiliations:** 1 Department of Psychology, University of Cincinnati, Cincinnati, OH USA; 2 Deparment of Psychological and Brain Sciences, University of Louisville, Louisville, KY USA

**Keywords:** Fear of negative evaluation, Anxiety sensitivity, Adverse childhood experiences, Adolescent young adults, College students

## Abstract

Fear of negative evaluation (FNE) impacts adolescent young adult (AYA) college students’ mental health through impaired academic and social performance. Adverse childhood experiences (ACEs) are prevalent among college students and may play a role in exacerbating FNE symptoms. Early life stressors are associated with anxiety sensitivity (AS) which may contribute to the development or worsening of FNE. We examined predictors of FNE among college students during the COVID-19 pandemic, hypothesizing that AS would mediate the relationship between ACEs and FNE. Participants were 192 students (*M*_*age*_=19.90, *SD* = 1.26; 66.7% female; 78.6% non-Hispanic White) from a public, midwestern university. Participants completed demographics, ACEs, AS, and FNE questionnaires during March and April of 2020. AS fully mediated the relation between ACEs and FNE. Students who experienced greater ACEs reported increased AS and FNE. Findings suggest AS may be a help prevent further psychological distress from FNE among students who have been impacted by childhood adversity.

## Introduction

Fear of negative evaluation (FNE), the belief and anxiety that one will be critically judged in social situations (Weeks et al., 2005; Yue & Jia, 2023), can impact college students’ wellbeing through impaired academic and social performance (Ghaedi et al., 2010). FNE is characterized by worry about being evaluated by others, distress over assumed negative evaluations, and expectations of being critically judged by others (Weeks et al., 2005). The college student population is primarily comprised of adolescents and young adults (AYAs), and their developmental stage is reflective of large cognitive, social, and academic demands as AYAs navigate the transition from adolescence to adulthood. Cognitive factors may predispose AYAs to experience FNE; AYAs’ emotion and reward centers of the brain develop earlier than the prefrontal cortex, making social rewards like seeking peer validation and opinions reinforcing (Abrams, 2023; Feng et al., 2022). Among AYAs, FNE is related to social anxiety (Fredrick & Luebbe, 2022), suicidal ideation (Preston et al., 2023), social media addiction (Ali et al., 2021), and academic stress and underachievement (Nonterah et al., 2015; Saddler & Buley, 1999). Targeting FNE may have downstream effects on AYA college students’ socioemotional wellbeing, mental health, and academic achievement.

Despite the importance of FNE, there is a paucity of research examining predictors of FNE in college student AYAs (Hazel et al., 2014). Previous research has identified that low self-esteem and feelings of inferiority around one’s appearance, academic abilities, and physical abilities predict FNE (Li et al., 2023; Sarwat Jahan Khanam & Fazeela Moghal, 2012; You et al., 2019; Yücens & Üzer, 2018). FNE has also been shown to mediate the relationship between self-esteem and social anxiety, and between the self-reinforcement construct of self-regulation and social anxiety (Kocovski & Endler, 2000).

One factor that has been related to other aspects of social comparison and anxiety, but has not been explored in relation to FNE, is adverse childhood experiences (ACEs). ACEs refer to a constellation of traumatic or stressful events occurring before the age of 18 that includes physical, emotional, sexual abuse/neglect and having a parent who is suffering from a mental illness, substance abuse, or domestic violence (Felitti et al., 1998; Karatekin & Hill, 2019). ACEs are prevalent among college students and are associated with impacts on health, behavior, and life success, such as increased risk for anxiety, suicide attempts, alcohol and drug misuse, reduced graduation rates and lower academic achievement (Centers for Disease Control and Prevention, 2019; Dube et al., 2002; Karatekin, 2018; Poole et al., 2017). A recent systematic review reported that 17.5–76.2% of college students endorsed at least one ACE and 0.3–24.6% endorsed four or more ACES (Schwartz et al., 2023).

Previous studies report that child maltreatment, particularly childhood emotional abuse and neglect, is associated with greater risk for the development of social problems and anxiety in adulthood (Bruce et al., 2012; Brühl et al., 2019; Kuo et al., 2011; Lin & Tsai, 2020). However, this prior research does not parse social anxiety into its components, nor evaluate the connection between ACEs and FNE, specifically. To our knowledge, few studies have specifically examined the association between ACEs and FNE (Beth, 1999; Lucero et al., 2022). The few studies examining the association between abuse and FNE have typically used assessments that examine an individual’s general abuse or trauma history rather than examining the relation between ACEs specifically and FNE. One study demonstrated positive associations between abuse and FNE (Beth, 1999), and another found that childhood abuse has been shown to be a predictor of mood disorders via FNE (Lucero et al., 2022).

Another possible predictor of FNE is anxiety sensitivity (AS), which is a heightened awareness of one’s anxiety symptoms and the belief that these anxiety symptoms are harmful (Reiss et al., 1986). Individuals with elevated AS fear the somatic symptoms of anxiety and experience physical, cognitive, and social concerns related to these fears (Taylor et al., 2007). AS is commonly linked with social anxiety, with several studies documenting AS as a predictor of social anxiety symptoms (Allan et al., 2017, 2018; Barlow, 2004; Brooke & Intrieri, 2023; Khakpoor et al., 2019). ACEs have also been identified as contributors to the development of AS (Zavos et al., 2012), and childhood maltreatment is well documented to be associated with AS (Amarneh et al., 2023; King et al., 2020; Martin et al., 2014; McLaughlin & Hatzenbuehler, 2009).

There is a current AYA mental health crisis following the COVID-19 pandemic (Rico et al., 2022), making it critical to understand and support this population’s mental health. During the pandemic, AYA mental health worsened; over 44% of AYAs reported persistent feelings of sadness, almost 20% seriously considered suicide, 9% attempted suicide, and many experienced a significant increase in anxiety, particularly social anxiety, resulting from the social isolation and academic disruption of the pandemic and subsequent shutdown (De Figueiredo et al., 2021; Jefferies & Ungar, 2020; Jones et al., 2021; Magson et al., 2021; Rico et al., 2022; Roche et al., 2022; Wang et al., 2020). AYA students attending college appeared to be particularly affected. The American Psychological Association published a large, comparative cohort study examining college student mental health pre- and post-pandemic (Nails et al., 2023). Findings suggest that the COVID-19 pandemic drastically increased college students’ anxiety and depression (Nails et al., 2023). These concerns are highly associated with FNE (Fredrick & Luebbe, 2022; Preston et al., 2023), and previous research suggests that targeting FNE could target these problems. This delineates a need to understand what predicts FNE in order to support college students’ wellbeing by safeguarding against downstream mental health concerns associated with FNE.

The current study examined predictors of FNE among AYA college students during the COVID-19 pandemic; this study is the first to our knowledge to examine AS and ACEs as correlates of FNE. We hypothesized that ACEs would be positively associated with FNE, and that this relationship would be mediated by AS, given AS is frequently conceptualized as a transdiagnostic mechanism linking two psychological constructs (Amarneh et al., 2023; King et al., 2020).

## Methods

### Participants

Participants were undergraduate students attending a large, public university in the Midwestern United States. Participants were recruited through Sona Systems, an online system utilized by the institution that provides course credits for students enrolled primarily in introductory psychology courses. Data for this study were collected in the context of a larger study. Study inclusion criteria were: (1) age *≥* at least 18 years, (2) currently enrolled at the university, and (3) not currently pregnant, an outcome in the larger study.

Data collection efforts took place during the early months of the COVID-19 shutdowns from March 2020 to April 2020. A total of 206 students consented to participate. To track valid responses, a validity question was utilized, asking participants to indicate if they responded to the questionnaires honestly. When accounting for responses to the validity question and completion of questionnaires measuring primary outcome variables, 14 participants were excluded from analyses, leaving a final sample of 192 students.

### Measures

#### Demographics

Participants completed a questionnaire created for the current study to assess demographic information (i.e., age, gender, ethnicity, race, marital status, living arrangements, and employment status).

#### Adverse Childhood Experiences

Exposure to ACEs was measured using the Adverse Childhood Experiences Questionnaire (ACE-Q; Felitti et al., 1998). Participants endorse whether they experienced 10 adverse experiences before the age of 18, including abuse (i.e., emotional, physical, sexual), neglect (i.e., physical, emotional), and household dysfunction (i.e., household substance abuse, mental illness, and incarceration, domestic violence against mother/stepmother, separation/divorce). A total score is calculated with higher scores representing more exposures. Previous research has supported the internal consistency of the ACE-Q, with a Cronbach’s alpha of 0.88 in a clinical and community sample (Murphy et al., 2014).

#### Anxiety Sensitivity

AS was measured using the Anxiety Sensitivity Index 3 (ASI-3; Taylor et al., 2007) an 18-item self-report measure assessing participants’ concerns of potential negative consequences of anxiety-related symptoms. Participants rated their beliefs about the consequences of symptoms associated with anxious arousal from 0 (very little) to 4 (very much). This measure examines three aspects of anxiety arousal: physical symptoms (e.g., It scares me when my heart beats rapidly), cognitive symptoms (e.g., It scares me when I am unable to keep my mind on a task), and social concerns (e.g., When I tremble in the presence of others, I fear what people might think of me). Items were summed to provide a total score ranging from 0 to 72. ASI-3 total scores between 0 and 16 suggest low AS, scores of 17–22 suggest moderate-to-high anxiety sensitivity, and scores 23 and above indicate high levels of AS (Allan et al., 2014). The ASI-3 has been found to be a psychometrically sound and valid measure of AS with high internal consistency (i.e., Cronbach *α* = 0.93; Taylor et al., 2007; Wheaton et al., 2012).

#### Fear of Negative Evaluation

FNE was measured using the Brief Fear of Negative Evaluation Scale (BFNE; Leary, 1983) consisting of 12 self-report questions on social anxiety and fear associated with criticism (e.g., I am afraid that people will find fault with me; Other people’s opinions do not bother me), which are rated from 1 (not at all characteristic of me) to 5 (extremely characteristic of me). Items are summed for a total score ranging from 12 to 60; high scores indicate greater fears of negative evaluation. The BFNE is a valid and reliable measure with a high inter-item reliability (Cronbach’s α = 0.90; Leary, 1983).

### Procedure

All study procedures were approved by the Institutional Review Board at the authors’ institution. Participants accessed and completed study measures through a one-time online survey using REDCap (i.e., Research Electronic Data Capture), a secure web application for conducting online studies. Study information was displayed on Sona Systems (described in Participants section). If interested in participating, students clicked on a link that directed them to a detailed study information page, where they then consented by clicking on a link to the survey. Participants completed a survey for inclusion criteria (described in Participants section) and, if eligible, were directed to study measures. Upon completion of the survey, each participant received research credits through the Sona Systems website.

### Data Analysis

Utilizing SPSS (IBM SPSS Statistics 29), data were cleaned, evaluated for normality and outliers, and analyzed. Descriptive statistics were used to describe sample characteristics, and key demographics (i.e., gender, race, ethnicity) were tested as potential covariates predicting FNE. Gender was the only significant covariate (*p* = .04) and was included in the mediation model. PROCESS (Hayes SPSS PROCESS Macro Version 4) was used to test for the mediating effect of AS on the relationship between ACEs and FNE. Total scores for ACEs, AS, and FNE were included in the model. Due to data not being normally distributed, bootstrapping analyses were utilized (Efron, 1992). All significance levels were set at *p* < .05.

## Results

### Descriptive Statistics

Participants primarily identified as female (*n* = 128, 66.7%), White (*n* = 151, 78.6%), and non-Hispanic (*n* = 184, 95.8%). The mean age of participants was 19.90 years (*SD* = 1.26). See Table 1 for related participant demographics. Participants reported moderate-to-high AS, with an average score of 19.40 (*SD* = 14.50). Participants scored an average of 38.64 (*SD* = 10.05) on the BFNE. Participants on average reported 1.73 ACEs (*SD* = 1.98) out of 10, with 60.9% of the sample having experienced at least one ACE (*n* = 117). Most participants reported having experienced emotional abuse as a child (*n* = 63; 32.8%), parental divorce (*n* = 55, 28.6%), and household mental illness (*n* = 50,26%).

Total scores for AS, FNE, and ACEs were positively correlated with one another. See Table 2. 

**Table 1:** Participant demographic information

	*N* (%)
Gender	
Female	128 (66.7)
Male	61 (31.8)
Transgender and non-binary	3 (1.6)
Race	
Caucasian/White	151 (78.6)
African American/Black	11 (5.7)
Asian	16 (8.3)
Multiracial/other race	14 (7.3)
Ethnicity	
Hispanic/Latino	7 (3.6)
Not Hispanic/Latino	184 (95.8)
Unknown	1 (0.5)
Marital Status	
Currently married	13 (6.8)
Not married, living with partner	9 (4.7)
Single, never married	170 (88.5)
Employment	
Employed full-time	3 (1.6)
Employed part-time	92 (47.9)
Not employed, looking for work	49 (25.5)
Not employed, not looking for work	47 (24.5)
Employed, on medical leave	1 (0.5)
Living arrangement	
On campus	76 (39.6)
Off-campus with parent(s)	62 (32.3)
Off-campus living alone	5 (2.6)
Off-campus with roommate(s)	45 (23.4)
Other living arrangements	4 (2.1)

Note. Demographic information was calculated with a final sample of 192 students

**Table 2:** Zero-order correlations among variables

	1	2	3
1. *AS*	-		
2. *FNE*	0.573**	-	
3. *ACEs*	0.368**	0.208**	-

Note. AS = total anxiety sensitivity score; FNE = total fear of negative evaluation score; ACES = total adverse childhood experiences score; *Correlation is significant at the 0.01 level (2-tailed); *p* < .001

### Mediation Model

**Fig. 1:**
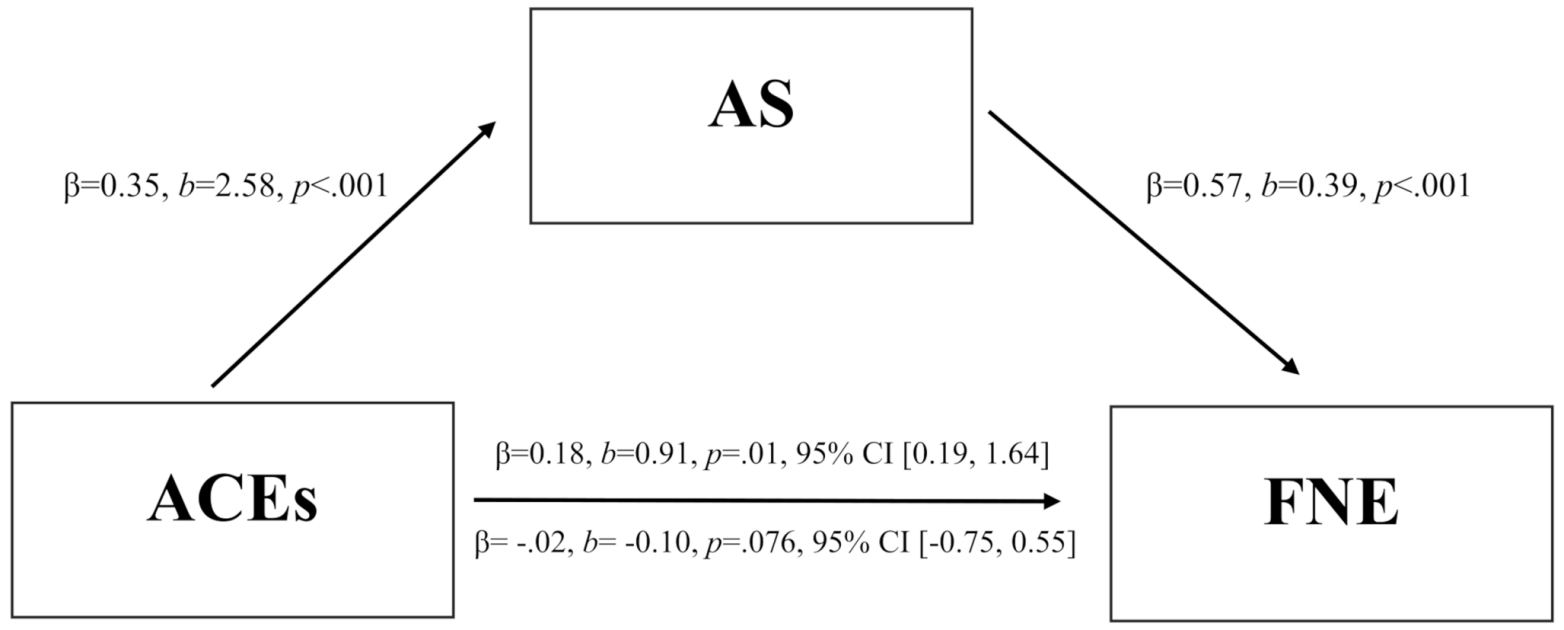
Mediation model. Note: Results of the mediation analyses, including standardized (β) and unstandardized (b) path coefficients. Coefficients above the arrow are estimates of the total effect; those below the arrow are estimates of the direct effect. ACEs = adverse childhood experiences, AS = anxiety sensitivity, FNE = fear of negative evaluation. Gender was initially a significant covariate for predicting fear of FNE (*p* = .04) but was not significant once it was included in the mediation model

A significant direct effect was found for ACEs on FNE (*b* = 2.58, *t* = 5.06, *p* < .001). The indirect effect of AS on the relationship between ACEs and FNE was significant (a*b = 1.01, CI95 = 0.59–1.50). AS completely accounted for the total effect of ACEs on FNE [PM= (1.01)/ (0.91)]. There was no significant direct effect between ACEs and FNE when controlling for AS (*b* = -0.10, *t* = -0.30 *p* = .76), suggesting full mediation. Gender was no longer significant once it was included in the mediation model. See Fig. 1.

## Discussion

The current study was the first to our knowledge to examine ACEs and AS as correlates of FNE among college students. Students who experienced more ACEs reported higher levels of AS, indicating a heightened fear of experiencing anxiety-related sensations. Additionally, greater ACEs were associated with increased FNE, reflecting a greater concern about being judged negatively in social situations. AD fully mediated the relationship between ACEs and FNE. These findings align with prior research that suggests childhood exposure to adverse experiences can have lasting effects on psychological functioning, contributing to the development of anxiety-related symptoms in young adulthood (Amarneh et al., 2023; King et al., 2020), while also being nuanced in the context of the COVID-19 pandemic.

AS completely accounted for the total effect of ACEs on FNE, suggesting that heightened FNE among participants with ACEs is largely driven by heightened sensitivity to anxiety symptoms. Further, heightened awareness of one’s own anxiety symptoms may lead to the development of fears and worries associated with perceptions of others. Individuals with heightened AS are more likely to be hypervigilant about their own physical, cognitive, and emotional responses to stressful events (Ngien & Hogan, 2023). In the context of a global pandemic, a period of increased stress, where social interactions were limited and often shifted to virtual platforms, this heightened self-focus may have become more pronounced (Ngien & Hogan, 2023). This is especially relevant considering the increase in online courses since the pandemic (Clary et al., 2022). More research is needed in this area to examine the effects of the pandemic on AS.

The link between AS and FNE in the context of experience of ACEs can be conceptualized as a cascade of events among AYAs. AS is significant during stressful events; AYAs with heightened AS may interpret these symptoms as negative and threatening, possibly exacerbating anxiety and contributing to an increasing cycle of heightened AS (Yue & Jia, 2023). Through increased awareness of anxiety symptoms and potential negative consequences of these symptoms, concern of external perceptions may increase as well. This progression is where FNE can foster; AYAs may worry that others will notice their anxiety-related sensations and interpret these as signs of incompetence and inadequacy (Weeks et al., 2005). Additionally, childhood maltreatment, particularly parental threatening behavior, is associated with greater AS in AYAs (Scher & Stein, 2003). Exposure to certain ACEs that involve uncontrolled parental behavior, such as emotional abuse or parental substance abuse, might dispose the child to a fear of their own symptoms of arousal and loss of control, further increasing the likelihood of developing anxiety symptoms and AS (Watt & Stewart, 2003).

### Clinical Implications

The findings of this study have several important clinical implications. FNE is a key diagnostic criterion for the diagnosis of social anxiety (American Psychiatric Association, 2013), and is associated with several other mental health conditions (e.g., social media addiction, and suicidality; Ali et al., 2021; Preston et al., 2023), academic stress, and underachievement (Nonterah et al., 2015; Saddler & Buley, 1999). Hence, understanding the connection of ACEs to FNE through AS allows for targeted, empirically supported treatments to alleviate later presence of greater psychological symptoms and subsequent harmful behaviors. Previous literature has supported that AS is responsive to interventions such as cognitive-behavioral treatments that utilize mindfulness techniques (Otto & Reilly-Harrington, 1999). Hence, treating AS could potentially alleviate the effects of ACEs on FNE, subsequently improving and preventing further psychological distress from FNE symptoms. Providing mental health resources that build on resilience, emotion regulation, psychoeducation, and effective coping strategies in tandem with traditional therapeutic approaches may aid in breaking the cycle of heightened AS leading to FNE in college students with ACEs (Allan et al., 2015; Asnaani et al., 2020; Dąbkowska et al., 2021). The mediating effect of AS on ACEs and FNE underscores the importance of a transdiagnostic approach to addressing mental health concerns in college students and the underlying mechanisms, ultimately improving the well-being of AYAs, particularly during challenging times. The study findings also highlight the importance of addressing AS in interventions that target FNE, particularly for students who have experienced childhood stress and trauma. Integrating interventions that target AS may be more effective in improving overall mental well-being than focusing solely on specific disorders. By targeting underlying mechanisms that cut across different psychological conditions, clinicians and educators may provide more comprehensive and efficient support to college students experiencing distress.

### Limitations

Although the current study is novel in assessing the relationship among FNE, AS, and ACEs among college students during the COVID-19 pandemic, it is essential to acknowledge several limitations. Data for this study was collected during the early months of the COVID-19 lockdown, a time marked by sudden changes to university policy and curriculum, and unpredictability from the status of the COVID-19 virus, followed by subsequent increases in mental health concerns among AYA student populations. Results should be interpreted in the context of this timeframe. Future research should examine the relationship between FNE, AS, and ACEs during a timeframe where COVID-19 does not restrict traditional academics, extracurricular activities, or social interaction to determine whether these findings are generalizable to AYAs outside the context of COVID-19.

Due to the cross-sectional design, causality cannot be determined, and relationships explored in this study can only be considered as associations. Future research should longitudinally examine the relation between ACEs and FNE and the role of AS in mediating this relationship over time. Additionally, the use of self-report to assess FNE and AS and retrospective-report of ACEs may have resulted in biased measurement of study constructs. Future research should utilize multiple methods and multiple raters, such as clinician or parent report of symptoms, to reduce the impact of bias.(Brenner & DeLamater, 2016) Additionally, current recommendations for measuring ACEs suggest using a dimensional approach that assesses individual exposure to each adverse experience rather than assessing ACEs as a binary (McLennan et al., 2020; Reidy et al., 2021).

The study’s sample was comprised of college students primarily identifying as female, non-Hispanic, and White from one university in the United States. This limits the generalizability of results to other diverse groups and samples of young adults who are not students. Future research should examine these variables within more diverse populations to determine whether current study findings can be replicated within racial and ethnic minoritized groups as well as non-student AYAs.

## Conclusions

The current study supports that AS is a critical component in the understanding of the relationship between ACEs and FNE among AYA college students during the early months of the COVID-19 pandemic. AS may be a potential factor that can be addressed via psychological interventions to alleviate the long-term consequences of ACEs on the development of fears relating to anxiety in college students. Therapeutic strategies to cope with symptoms of AS could alter the presentation of FNE in students who have been impacted by ACEs. Future studies using a longitudinal design to establish a causal relationship between ACEs, FNE, and AS are needed to further the literature on FNE among college students with histories of ACEs.
